# The role of embryo contact and focal adhesions during maternal recognition of pregnancy

**DOI:** 10.1371/journal.pone.0213322

**Published:** 2019-03-05

**Authors:** K. M. Klohonatz, L. C. Nulton, A. M. Hess, G. J. Bouma, J. E. Bruemmer

**Affiliations:** 1 Department of Animal Sciences, Colorado State University, Fort Collins, Colorado, United States of America; 2 Department of Statistics and Bioinformatics, Colorado State University, Fort Collins, Colorado, United States of America; 3 Department of Biomedical Sciences, Animal Reproduction and Biotechnology Laboratory, Colorado State University, Fort Collins, Colorado, United States of America; Houston Methodist Research Institute, UNITED STATES

## Abstract

Maternal recognition of pregnancy (MRP) in the mare is an unknown process. In a non-pregnant mare on day 14 post-ovulation (PO), prostaglandin F_2α_ (PGF) is secreted by the endometrium causing regression of the corpus luteum. Prior to day 14, MRP must occur in order to attenuate secretion of PGF. The embryo is mobile throughout the uterus due to uterine contractions from day of entry to day 14. It is unknown what signaling is occurring. Literature stated that infusing oil or placing a glass marble into the equine uterus prolongs luteal lifespan and that in non-pregnant mares, serum exosomes contain miRNA that are targeting the focal adhesion (FA) pathway. The hypothesis of this study is embryo contact with endometrium causes a change in abundance of focal adhesion molecules (FA) in the endometrium leading to decrease in PGF secretion. Mares (n = 3/day) were utilized in a cross-over design with each mare serving as a pregnant and non-pregnant (non-mated) control on days 9 and 11 PO. Mares were randomly assigned to collection day and endometrial samples and embryos were collected on the specified day. Biopsy samples were divided into five pieces, four for culture for 24 hours and one immediately snap frozen. Endometrial biopsies for culture were placed in an incubator with one of four treatments: [1] an embryo in contact on the luminal side of the endometrium, [2] beads in contact on the luminal side of the endometrium, [3] peanut oil in contact on the luminal side of the endometrium or [4] the endometrium by itself. Biopsies and culture medium were frozen for further analysis. RNA and protein were isolated from biopsies for PCR and Western blot analysis for FA. PGF assays were performed on culture medium to determine concentration of PGF. Statistics were performed using SAS (*P* ≤ 0.05 indicated significance). The presence of beads on day 9 impacted samples from pregnant mares more than non-pregnant mares and had very little impact on day 11. Presence of oil decreased FA in samples from pregnant mares on day 9. On day 11, oil decreased FA abundance in samples from non-pregnant mares. Embryo contact caused multiple changes in RNA and protein abundance in endometrium from both pregnant and non-pregnant mares. The PGF secretion after 24 hours with each treatment was also determined. On day 9, there was no change in PGF secretion compared to any treatments. On day 11, presence of peanut oil increased PGF secretion in samples from non-pregnant mares. In samples from non-pregnant mares, presence of an embryo decreased PGF secretion compared to control samples from non-pregnant mares. Results revealed that while beads and peanut oil may impact abundance of FA RNA and protein in endometrial samples, it does not appear to impact PGF secretion. Conversely, embryo contact for 24 hours with endometrium from a non-pregnant mare causes a decrease in PGF secretion. These results suggest that it is not just contact of any substance/object causing attenuation of PGF secretion, but the embryo itself is necessary to decrease PGF secretion.

## Introduction

Maternal recognition of pregnancy (MRP) refers to the mechanism by which endometrium identifies the presence of an embryo resulting in continued secretion of progesterone (P_4_) by the corpus luteum (CL) [1]. In non-pregnant mares on day 14 post-ovulation (PO), oxytocin is released from the endometrium into the uterine lumen, binding endometrial receptors, causing release of more oxytocin and production and release of prostaglandin F_2α_ (PGF)[2]. In pregnant mares, the conceptus enters the uterus on day 6, and by day 9 is surrounded by a glycoprotein rich capsule, a characteristic unique to the horse and rabbit [3] Once the conceptus is in the uterus, uterine contractions move the embryo throughout the uterus reaching peak mobility between days 11 and 14 PO [4,5]. This mobility is necessary to mitigate secretion of PGF. Embryo mobility ceases by day 16, indicating that MRP occurs between days 11 to 14 PO and functions in an antiluteolytic manner [4,6,7].

The CL must be protected from endometrial PGF in order to sustain P_4_ production [8–10]. Maternal recognition of pregnancy is vital to pregnancy success yet differs in the mare compared to other species. Interferon tau and estradiol are MRP signals in ruminants and pigs, respectively, but they do not have any impact on luteal function in the horse [11–14]. The equine conceptus secretes prostaglandin E_2_ on day 4, but it has no reported effect if infused into the uterus of non-pregnant mares [12]. Interestingly, when coconut or peanut oil was infused into the uterus of a NP mare on day 10 PO, luteostasis occurred, indicating that a component in these oils impacted the luteolytic pathway [15]. Literature also states that placing a glass ball, or marble, into the diestrous uterus of a mare will prolong the lifespan of the CL, suggesting it is physical contact onwith endometrium that prevents PGF secretion [16,17].

Exosomes, which are cell secreted vesicles, have been identified in equine serum [18]. Exosomes are capable of storing and transferring bioactive material, such as RNA and protein, between cells [19]. Recent literature has indicated that serum exosome content varied between pregnant and non-pregnant mares [20]. Specifically, it was found that differential miRNA in exosomes from non-pregnant mares potentially target the focal adhesion (FA) pathway [20]. Focal adhesions are macromolecular complexes that are comprised of heterodimeric transmembrane integrin receptors that regulate effects in extracellular matrices in endometrium [21]. It has also been reported that FA sense and transduce mechanical forces [22]. It has been suggested that mechanotransduction is the reason intrauterine devices resulted in luteal persistence in mares [16].

The present study was designed to test if presence of an embryo is necessary to attenuate PGF secretion, or if PGF secretion can be attenuated by oil or noncellular contact with endometrial epithelium. The hypothesis of this study is that contact of an embryo with equine endometrial epithelium will cause a change in FAM abundance and result in a decrease in PGF section.

## Materials and methods

### Care and management of mares

All horse use was approved by the Colorado State University Animal Care and Use Committee (Approval Number 13-4293A). Mares (n = 6) were housed in group pens at Colorado State University Equine Reproduction Laboratory (Fort Collins, CO) and maintained on a dry lot and fed grass-alfalfa mix with free choice mineral and salt supplement. Mares were used in a cross-over design in which each mare had a pregnant and non-pregnant (non-mated) cycle. Mares were teased with a stallion daily and transrectal ultrasonography was used to monitor to track their follicular development every other day. Once a follicle reached 35 mm in diameter, or greater, each mare was inseminated with 500 x 10^6^ progressively motile sperm from a single stallion with proven fertility. After insemination, mares were evaluated every day with transrectal ultrasonography and inseminated every other day until ovulation (day 0) was detected.

Mares were randomly assigned to one collection day (day 9 or 13) for their pregnant and non-pregnant cycles. On the assigned day, the mare was evaluated via transrectal ultrasonography to confirm pregnancy by the visualization of an embryonic vesicle. Endometrial samples were obtained non-surgically via a trans-cervical biopsy punch [23]. Embryos were collected via terminal uterine lavage on the same day as biopsy to be used in culture. After embryo and biopsy collection the mare received a luteolytic dose of prostaglandin F_2α_ (PGF; Estrumate, Merck Animal Health, 250 mcg per dose). The subsequent estrous cycle was utilized for the non-pregnant (non-mated) control cycle. Another herd of mares (n = 3) were monitored and bred with the same protocol in order to provide embryos to be cultured with endometrial samples from non-pregnant mares on corresponding days.

After endometrial biopsy samples were obtained, each sample was rinsed in DPBS/Modified 1X (Hyclone Laboratories, Logan, UT). Special care was taken to ensure the sample was not inverted in order to keep the luminal side of the endometrium facing upwards. Biopsy samples were cut into five pieces. Four pieces were then washed three times in incubation medium [(Medium 199 (Life Technologies, Carlsbad, CA) containing 5% antibiotic-antimycotic (Life Technologies, Carlsbad, CA)] prior to culture for 24 hours (explained in the next section). The fifth piece was immediately snap frozen in liquid nitrogen and transferred to -80°C.

### Endometrial biopsy culture

**Fig 1:**
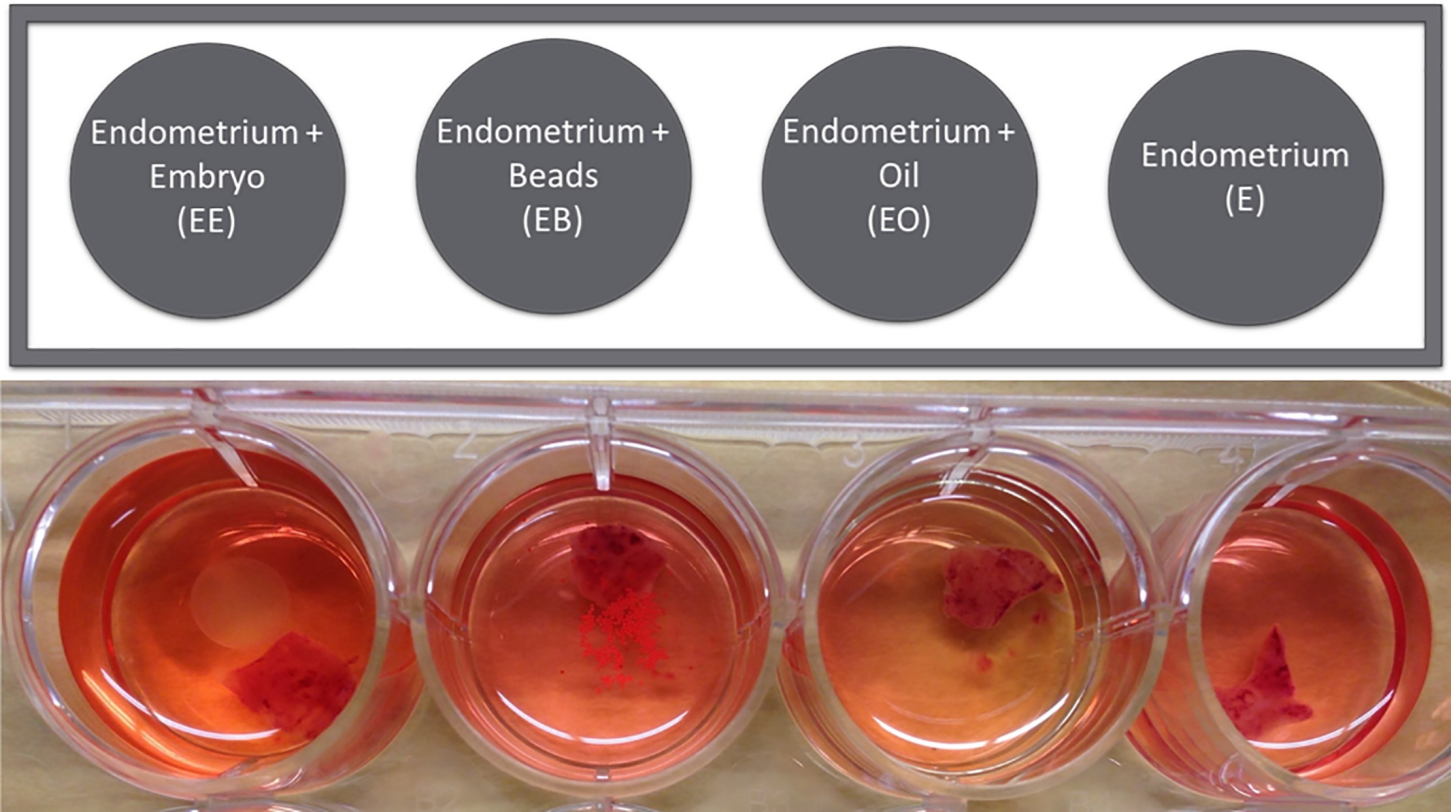
Endometrial culture layout. This figure represents the culture layout for each sample. Special care was taken to ensure the luminal side of the endometrium was in contact with the treatment for the entire 24 hours.

After endometrial biopsy and embryo collection, samples were immediately transferred to clean culture dishes for incubation for 24 hours with the corresponding treatment. Biopsy samples were cultured in one of four conditions: (1) direct contact with an embryo from the corresponding day (EE), (2) direct contact with plastic beads (utilized routinely to teach embryo flushing and transfer) (EB) (Cospheric, Product Number: UVPMS-BR-1-5, Santa Barbara, CA), (3) direct contact with peanut oil (EO) and (4) endometrial biopsy alone (control)(E-). Fig 1 contains a diagram of the culture method. Tissues were incubated in a humidified atmosphere at 5% CO_2_, 95% air at 37°C according to Watson and Sertich 1989 [24] for 24 hours. After 24 hours endometrial biopsies and their corresponding medium were immediately placed at -80°C until further analysis.

### RNA isolation and quantification

Total RNA was isolated from endometrial biopsies, both cultured and snap frozen, using TRI Reagent (Molecular Research Center, Cincinnati, OH). Frozen tissue was homogenized in TRI Reagent and left at room temperature for ten minutes. After the incubation, chloroform was added to the homogenate, vortexed and incubated for an additional eight minutes at room temperature. The sample was then centrifuged at 13,200 revolutions per minute (RPM; 16,100 x g) for 15 minutes in order to separate the sample into three distinct phases (RNA, DNA and protein). The top, aqueous phase RNA phase was transferred to a new 1.7 mL Eppendorf tube for RNA isolation. DNA and protein phases were frozen at -80°C until further analysis.

RNA was isolated from samples utilizing RNeasy Mini Kit (Qiagen, Valencia, CA). The protocol was followed according to manufacturer’s recommendations. Following isolation, all samples were treated with an RNase-Free DNase set (Qiagen, Valencia, CA) to remove any DNA contamination. RNA purity and quantification were assessed using the NanoDrop Spectrophotometer ND-1000 (Thermo Scientific, Wilmington, DE). Samples were used for PCR analysis if they had 260/280 and 260/230 values above 1.7.

### Real time quantitative polymerase chain reaction

The following genes were selected for evaluation within the endometrium based upon literature and previous data in our laboratory: *ACTN1*, *ACTN2*, *ACTN3*, *ACTN4*, *AKT3*, *BCL-2*, *CAV1*, *CCND1*, *ITGA10*, *ITGA4*, *ITGA5*, *ITGA6*, *ITGAV*, *ITGAX*, *ITGB1*, *ITGB3*, *PAK6*, *PTGS2*, *PTK2 (FAK)*, *RAF1*, *SLCO2A1* and *TLN1* (20, 21). Equine specific forward and reverse primers were designed using Primer3 (http://primer3.wi.mit.edu/). Primers were designed specifically to have a product size between 115–135 bp, a primer length between 19–27 bp, a primer Tm between 60–65°C, and a GC% content between 40–60. The genes, designed primer sequences and amplicon length for each of the genes can be found in S1 Table. Prior to PCR analysis, primer specificity, via DNA sequencing of PCR products was evaluated. PCR analysis for specificity was performed using an endometrial cDNA pool and GoTaq (Promega, Madison, WI) following the manufacturer’s protocol. PCR products were electrophoresed on a 2% agarose gel to confirm presence of an amplicon with the expected size. The product band was excised from the gel for DNA isolation with Qiaquick Gel Extraction (Qiagen, Valencia, CA). Isolated DNA was then sent to the University of California-Davis DNA Sequencing Facility, Davis, CA, to be sequenced. The resulting sequences were confirmed using NCBI BLAST for gene specificity.

For qRT-PCR analysis, total RNA was processed for reverse transcription using iScript cDNA Synthesis (Bio-Rad, Hercules, CA). 1 μg of total RNA was added to each reverse transcription reaction with 4 μL of 5x iScript reaction mix, 1 μL of iScript reverse transcriptase, and nuclease-free water to reach a total reaction volume of 20 μL. Reverse transcription was performed following the manufacturer’s specifications with 5 minutes at 25°C, 30 minutes at 42°C, 5 minutes at 85°C, and then holding at 4°C for immediate use as cDNA template in qRT-PCR. For each real time PCR reaction 5 μL of SsoAdvanced SYBR Green Supermix (Bio-Rad, Hercules, CA) was added to 2.5 μL of nuclease-free water, 1 μL of cDNA at a concentration of 50 ng/μL, and 1.5 μL of primer mix at a concentration of 10 μM to reach a final volume of 10 μL. Samples were loaded into 384 well LightCycler 480 plates (Roche, Indianapolis, IN) and analyzed in duplicate using a LightCycler 480 PCR System (Roche, Indianapolis, IN). Real Time PCR cycle conditions were per manufacturer’s protocol; 30 seconds at 95°C, and 40 cycles of denaturing at 95°C for 5 seconds and annealing and extension at 60°C for 30 seconds. PCR analysis followed by melt peak analysis occurring at 0.5°C increments from 65–95°C, holding for 2 seconds at each increment. Cq values were normalized to the geometric mean of *GAPDH*, *TUBA1B*, *B2M* and *ACTB* by subtracting the geometric mean from the Cq value (Microsoft Excel). These normalized values were used for the statistical analyses. For theses analyses, the treated sample was compared to the endometrial sample from the same day cultured by itself (control). Statistical analysis was performed with SAS 9.4 (SAS Institute Inc.). Proc Mixed was used to fit a mixed model separately by gene. Fixed effects included day (9 or 11), pregnancy status (pregnant or non-pregnant) and treatment (EE, EB, EO, E) plus all interactions. Horse ID and horse ID by pregnancy status were included as random effects to account for the repeated measures design. Tukey adjusted pairwise comparisons were used and significance was assessed at *P* ≤ 0.05.

### Protein isolation and quantification

Protein was isolated from all endometrial tissue utilizing RIPA buffer containing nuclease-free water, Tris pH 8.0, NaCl, glycerol, Nonidet P-40, SDS, deoxychlorate, ethylenediamine tetraacetic acid, HCl, 0.01% proteinase inhibitor PIC, and 0.05% PMSF. Frozen tissue samples were homogenized in RIPA lysis buffer while on ice, then sonicated on ice for 30 seconds and centrifuged at 9,300 RPM (10,000 x g) at 4°C for 10 minutes. The supernatant was placed into a new tube and PIC and PMSF were added. The protein content in samples was quantified using Pierce BCA Protein Assay Kit (Thermo Scientific, Wilmington, DE) following the manufacturer’s protocol. Briefly, standards were prepared using manufacturer provided BSA at 2.0 mg/mL. Working reagent utilizing manufacturer’s reagents A and B was prepared. A microplate was loaded with 25 μL of standard or sample and 200 μL of working reagent and the microplate was incubated at 37°C for 30 minutes. Samples were quantified using the Synergy 2 Multi-Mode Microplate Reader (Biotek, Winooski, VT). Sample concentrations were determined based on a standard curve prepared with the standards.

### Western blot analysis

Western blots were performed for proteins of interest based upon PCR results for all cultured and snap frozen samples. Western blots were normalized to Cytochrome C (primary antibody: sc-7159, rabbit polyclonal, Santa Cruz Biotechnologies, Santa Cruz, CA; secondary antibody: ab6721, goat anti-rabbit IgG with HRP, Abcam, San Francisco, CA) and all gels were run on a 4–15% gradient gel (Bio-Rad Laboratories, Hercules, CA, catalog number 4561086). Proteins of interest were (Table 1): FAK (1:200 primary antibody: sc-558, rabbit polyclonal, Santa Cruz Biotechnologies, Santa Cruz, CA; 1:2000 secondary antibody: ab6721, goat anti-rabbit IgG with HRP, Abcam, San Francisco, CA), p-FAK (1:200 primary antibody: sc-16563-R, rabbit polyclonal, Santa Cruz Biotechnologies, Santa Cruz, CA; 1:2000 secondary antibody: ab6721, goat anti-rabbit IgG with HRP, Abcam, San Francisco, CA), Integrin β3 (1:200 primary antibody: sc-6627, goat polyclonal, Santa Cruz Biotechnologies, Santa Cruz, CA; 1:2000 secondary antibody: sc-2345, mouse anti-goat IgG with HRP, Santa Cruz Biotechnologies, Santa Cruz, CA), α-actinin (1:200 primary antibody: sc-7453, goat polyclonal, Santa Cruz Biotechnologies, Santa Cruz, CA; 1:2000 secondary antibody: sc-2345, mouse anti-goat IgG with HRP, Santa Cruz Biotechnologies, Santa Cruz, CA), PAK6 (1:500 primary antibody: ab37749, rabbit polyclonal, Abcam, San Francisco, CA; 1:2000 secondary antibody: ab6721, goat anti-rabbit IgG with HRP, Abcam, San Francisco, CA), CCND1 (1:200 primary antibody: ab7958, rabbit polyclonal, Abcam, San Francisco, CA; 1:2000 secondary antibody: ab6721, goat anti-rabbit IgG with HRP, Abcam, San Francisco, CA), BCL-2 (1:200 primary antibody: sc-492, goat polyclonal, Santa Cruz Biotechnologies, Santa Cruz, CA; 1:2000 secondary antibody: sc-2345, mouse anti-goat IgG with HRP, Santa Cruz Biotechnologies, Santa Cruz, CA) and CAV1 (1:200 primary antibody: sc-894, rabbit polyclonal, Santa Cruz Biotechnologies, Santa Cruz, CA; 1:2000 secondary antibody: ab6721, goat anti-rabbit IgG with HRP, Abcam, San Francisco, CA).

**Table 1:** Proteins and antibody information used for western blot analyses.

Protein	Primary Antibody				Secondary Antibody			
	Dilution	Type	Company	Catalog Number	Dilution	Type	Company	Catalog Number
Cytochrome C	1:200	Rabbit Polyclonal	Santa Cruz Biotechnologies	sc-7159	1:2000	Goat anti-rabbit IgG with HRP	Abcam	ab6721
FAK	1:200	Rabbit Polyclonal	Santa Cruz Biotechnologies	sc-558	1:2000	Goat anti-rabbit IgG with HRP	Abcam	ab6721
p-FAK	1:200	Rabbit Polyclonal	Santa Cruz Biotechnologies	sc-16563-R	1:2000	Goat anti-rabbit IgG with HRP	Abcam	ab6721
Integrin β3	1:200	Goat Polyclonal	Santa Cruz Biotechnologies	sc-6627	1:2000	Mouse anti-goat IgG with HRP	Santa Cruz Biotechnologies	sc-2345
α-actinin	1:200	Goat Polyclonal	Santa Cruz Biotechnologies	sc-7453	1:2000	Mouse anti-goat IgG with HRP	Santa Cruz Biotechnologies	sc-2345
PAK6	1:500	Rabbit Polyclonal	Abcam	ab37749	1:2000	Goat anti-rabbit IgG with HRP	Abcam	ab6721
CCND1	1:200	Rabbit Polyclonal	Abcam	ab7958	1:2000	Goat anti-rabbit IgG with HRP	Abcam	ab6721
BCl-2	1:200	Goat Polyclonal	Santa Cruz Biotechnologies	sc-492	1:2000	Mouse anti-goat IgG with HRP	Santa Cruz Biotechnologies	sc-2345
CAV1	1:200	Rabbit Polyclonal	Santa Cruz Biotechnologies	sc-894	1:2000	Goat anti-rabbit IgG with HRP	Abcam	ab6721

For each Western blot, 20 μg of endometrial protein was loaded into each well. Samples were incubated with a 4:1 6x buffer to DTT mix for 10 minutes at 90°C. Samples were then transferred to wells and run for 35 min at 200 V and transferred to nitrocellulose membranes (Bio-Rad Laboratories, Hercules, CA, Catalog #1620213) for 1 hour at 100 V at 4°C. Membranes were blocked in 5% blocking buffer (5% non-fat dried milk in 1X TBST) for 1 hour at room temperature and washed with 1X TBST. Membranes were incubated with primary antibody overnight at 4°C on a rocker. The next day membranes were washed three times in 1X TBST for 5 min and incubated with horseradish peroxidase conjugated secondary antibody for one hour at room temperature. Membranes were washed three times in 1X TBST and ECL Plus Western Blotting Detection Reagent (GE Healthcare Life Sciences, Pittsburgh, PA, Catalog #RPN2232) was added for 5 minutes. Membranes were imaged for analysis on a Molecular Imager ChemiDoc XRS+ System (Bio-Rad, Hercules, CA).

For theses analyses, the treated sample was compared to the endometrial sample from the same day cultured by itself (control). Statistical analysis was performed with SAS 9.4 (SAS Institute Inc). Proc Mixed was used to fit a mixed model separately by gene. Fixed effects included day (9 or 11), pregnancy status (pregnant or non-pregnant) and treatment (EE, EB, EO, E) plus all interactions. Horse ID and horse ID by pregnancy status were included as random effects to account for the repeated measures design. Tukey adjusted pairwise comparisons were used and significance was assessed at *P* ≤ 0.05.

### Prostaglandin F_2α_ hormone assay

In order to quantify the amount of PGF secreted by the endometrium after culture, a PGF_2α_ EIA kit from Enzo Life Sciences was utilized (Enzo Life Sciences, Farmingdale, NY, catalog number ADI-901-069). This is a competitive immunoassay utilizing a polyclonal antibody to PGF. Briefly, standards with known concentrations of PGF were prepared utilizing incubation medium. 100 μL of standards and samples were pipetted into the corresponding wells. Samples were evaluated in duplicate. Assay buffer, conjugate, and antibody were added to each well. After a two-hour incubation at room temperature, the wells were washed with wash solution. Substrate solution was added to each well and incubated for 45 minutes at room temperature. After stop solution was added to each well the plate was read at an optical density of 405 nm.

Unknown concentrations were determined utilizing a four-parameter logistic fit. Once the concentration was determined, it was adjusted based upon an adjustment factor equal to the weight of the original tissue divided by the mL of medium. There were varying amounts of medium in the wells due to the need to ensure the entire embryo was covered. For theses analyses, the treated sample was compared to the endometrial sample from the same day cultured by itself (control). Concentrations were analyzed for statistical significance using SAS 9.4 (SAS Institute Inc.). Proc Mixed was used to fit a mixed model separately by gene. Fixed effects included day (9 or 11), pregnancy status (pregnant or non-pregnant) and treatment (EE, EB, EO, E) plus all interactions. Horse ID and horse ID by pregnancy status were included as random effects to account for the repeated measures design. Tukey adjusted pairwise comparisons were used and significance was assessed at *P* ≤ 0.05.

## Results

### Focal adhesion molecules in snap frozen endometrium

**Fig 2:**
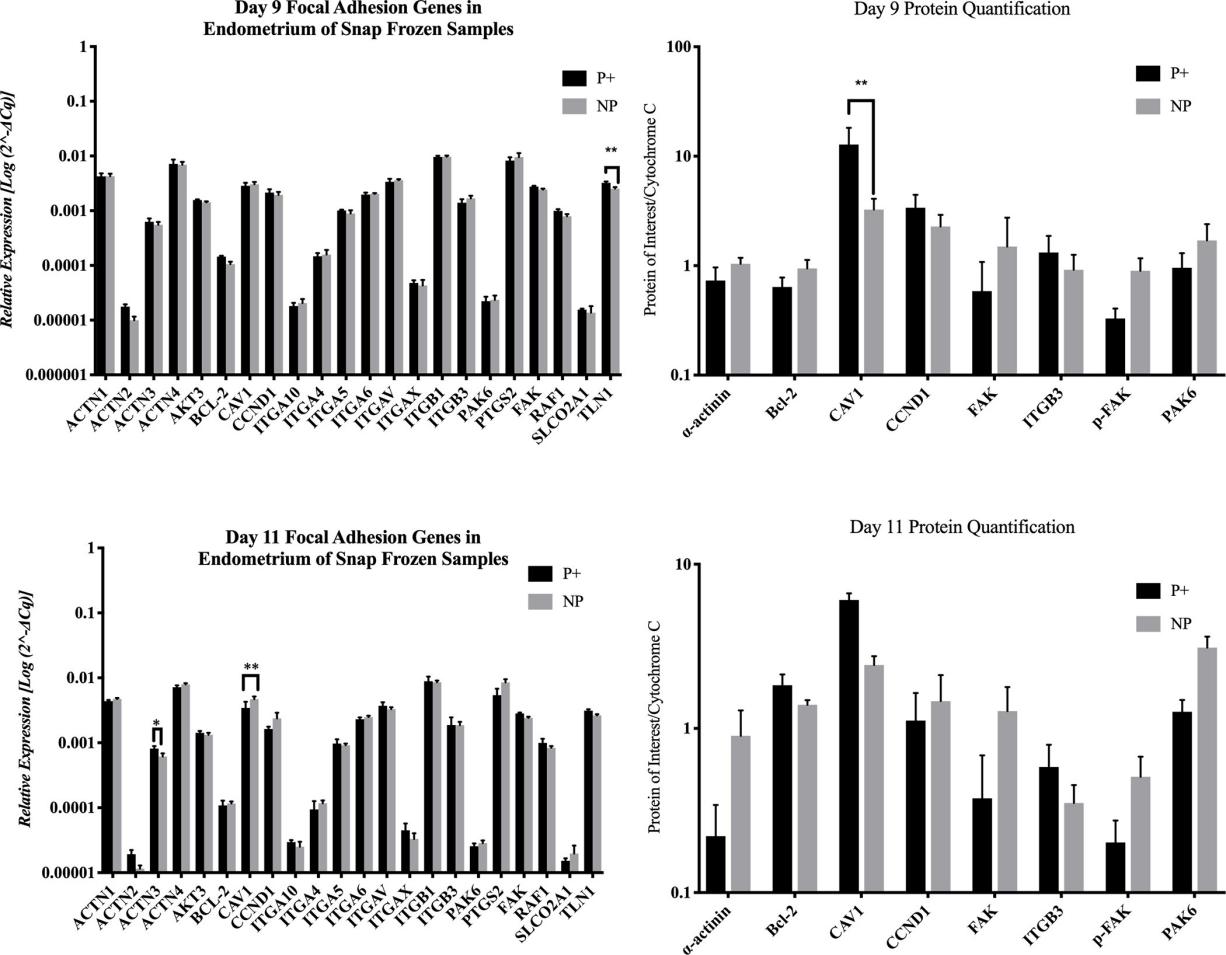
Endometrial gene and protein abundance in snap frozen endometrial samples from pregnant (P+) and non-pregnant (NP) mares. This figure shows the gene and protein abundance of targets for this study. * = *P* ≤ 0.05, ** = *P* ≤ 0.01 and *** = *P* ≤ 0.001. Graphs are mean ± SEM.

There were very few transcripts differentially expressed on days 9 or 11. On day 9, *TLN1* was increased (*P* = 0.011) in endometrial samples from pregnant mares. Also, in samples from pregnant mares on day 9, CAV1 was more abundant (*P* = 0.004). On day 11, *ACTN3* was increased (*P* = 0.050) in endometrial samples from pregnant mares and *CAV1* was increased (*P* = 0.011) in endometrial samples from non-pregnant mares. Fig 2 shows the transcript levels on days 9 and 11. There were no differences identified in protein abundance on day 11 (Fig 2).

### Endometrium co-cultured with mechanical pressure

**Fig 3:**
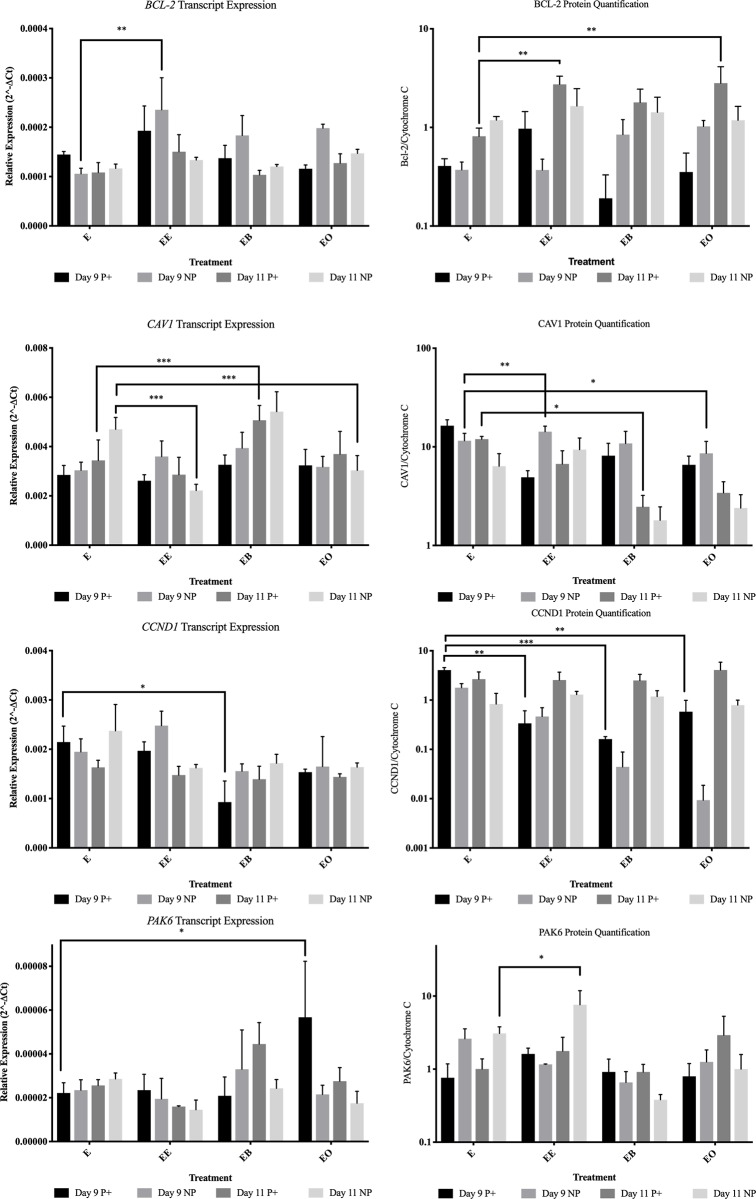
Gene and protein abundance for Bcl-2, CAV1, CCND1 and PAK6 for all treatments in endometrial samples from pregnant (P+) and non-pregnant (NP) mares. This figure contains all of the gene expression and protein quantification data for Bcl-2, CAV1, CCND1 and PAK6. E = no treatment (control); EE = endometrium with an embryo in contact with the luminal side of the biopsy sample; EB = endometrium with beads in contact with the luminal side of the biopsy sample; EO = endometrium with beads in contact with the luminal side of the biopsy sample. * = *P* ≤ 0.05, ** = *P* ≤ 0.01 and *** = *P* ≤ 0.001. Graphs are mean ± SEM.

**Fig 4:**
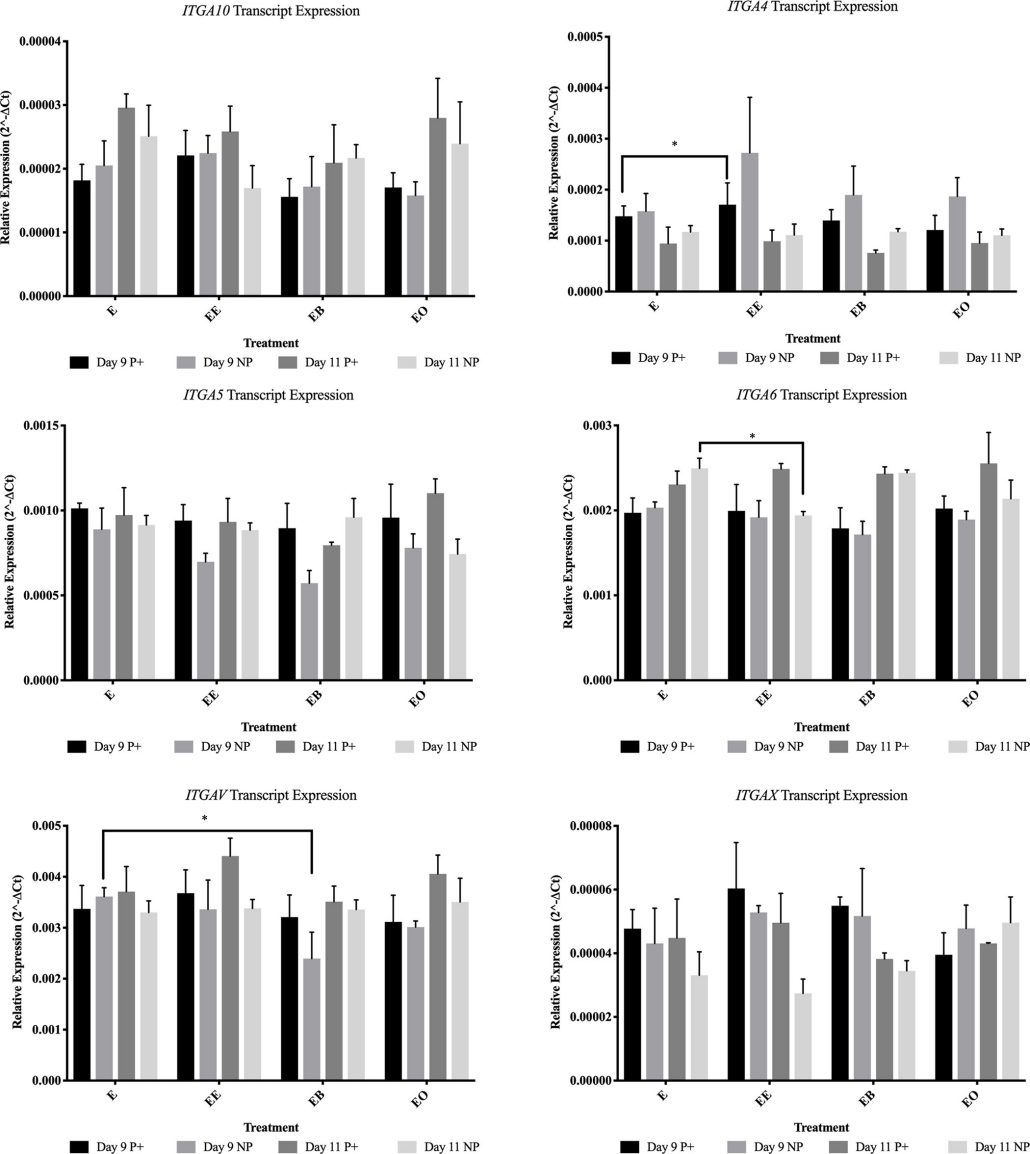
Gene abundance for α-integrins for all treatments in endometrial samples from pregnant (P+) and non-pregnant (NP) mares. This figure contains all of the protein and gene expression data that was analyzed in this project. E = no treatment (control); EE = endometrium with an embryo in contact with the luminal side of the biopsy sample; EB = endometrium with beads in contact with the luminal side of the biopsy sample; EO = endometrium with beads in contact with the luminal side of the biopsy sample. * = *P* ≤ 0.05, ** = *P* ≤ 0.01 and *** = *P* ≤ 0.001. Graphs are mean ± SEM.

**Fig 5:**
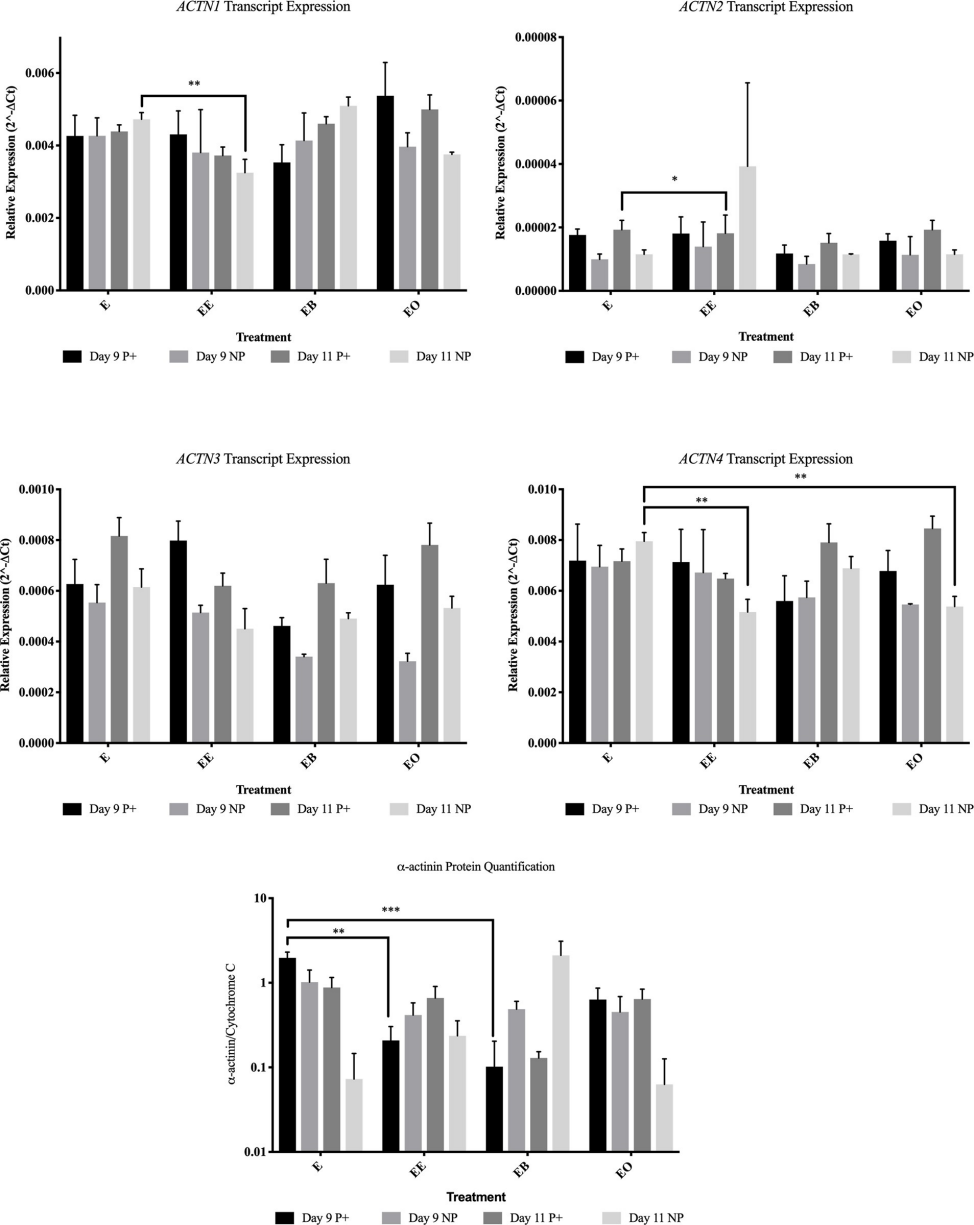
Actin genes and protein abundance for all treatments in endometrial samples from pregnant (P+) and non-pregnant (NP) mares. This figure contains all of the gene expression and protein quantification for ACTN1-4 and α-ACTININ protein data. E = no treatment (control); EE = endometrium with an embryo in contact with the luminal side of the biopsy sample; EB = endometrium with beads in contact with the luminal side of the biopsy sample; EO = endometrium with beads in contact with the luminal side of the biopsy sample. * = *P* ≤ 0.05, ** = *P* ≤ 0.01 and *** = *P* ≤ 0.001. Graphs are mean ± SEM.

**Fig 6:**
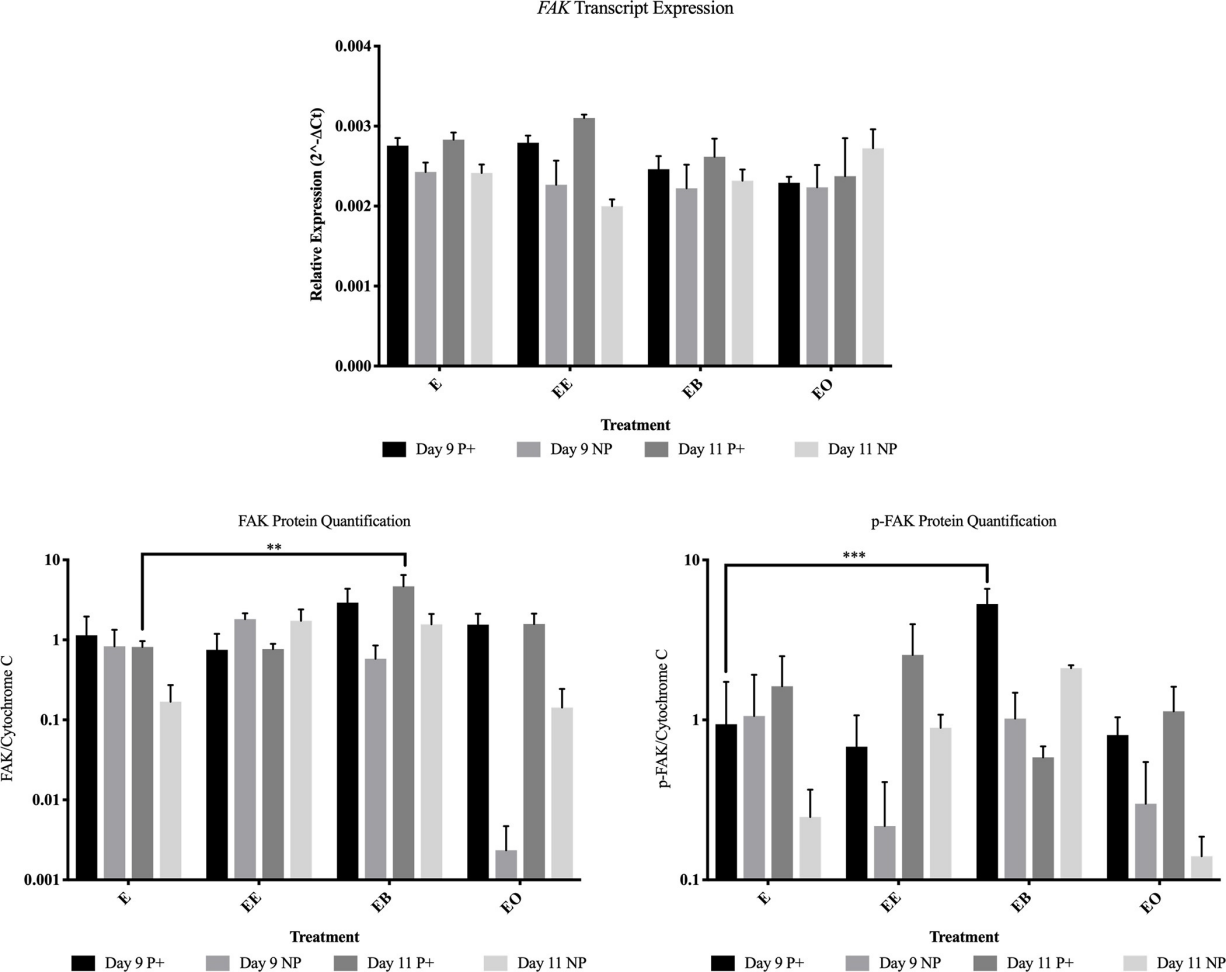
FAK and p-FAK expression and protein abundance for all treatments in endometrial samples from pregnant (P+) and non-pregnant (NP) mares. This figure contains the gene and protein levels for FAK and p-FAK in the endometrium on days 9 and 11. E = no treatment (control); EE = endometrium with an embryo in contact with the luminal side of the biopsy sample; EB = endometrium with beads in contact with the luminal side of the biopsy sample; EO = endometrium with beads in contact with the luminal side of the biopsy sample. * = *P* ≤ 0.05, ** = *P* ≤ 0.01 and *** = *P* ≤ 0.001. Graphs are mean ± SEM.

On day 9 from endometrial samples collected from pregnant mares, *CCND1* was more abundant (*P* = 0.015) in control tissue (Fig 3). In endometrial samples collected from non-pregnant mares, *ITGAV* was more abundant (*P* = 0.013) in control tissue (Fig 4). When evaluating protein levels, the samples from pregnant mares had different protein abundance. α-ACTININ (Fig 5) and CCND1 (Fig 3) were more abundant (*P* = 0.001 and *P* = 0.001 respectively) in control tissue and p-FAK was more abundant (*P* < 0.001) in endometrial samples co-cultured with beads (Fig 6).

On day 11, *CAV1* was more abundant (*P* = 0.001) in samples from pregnant mares with mechanical pressure on the endometrium. FAK was more abundant (*P* = 0.004) in samples with mechanical force from pregnant mares and CAV1 was more abundant (*P* = 0.030) in control samples from pregnant mares (Figs 3 and 6).

### Endometrium co-cultured with peanut oil

On day 9, no transcript or protein levels in endometrial samples from non-pregnant mares were affected by co-culture with oil. *PAK6* was more abundant (*P* = 0.012) in samples from pregnant mares co-cultured with peanut oil. Only protein levels in samples from pregnant mares were impacted by the presence of oil. CAV1 (*P* = 0.024) and CCND1 (*P* = 0.005) were all in higher abundance in control samples compared to the samples co-cultured with peanut oil (Fig 3).

On day 11, transcript differences were only identified in samples from non-pregnant mares. *ACTN4* (*P* = 0.011) and *CAV1* (*P* = 0.001) were more abundant in control tissue samples compared to samples co-cultured with peanut oil (Figs 3 and 5). There were no changes in protein abundance in endometrial samples collected from non-pregnant mares. BCL-2 was the only protein that was higher in abundance (*P* = 0.007) in the presence of oil in samples collected from pregnant mares (Fig 3).

### Endometrium co-cultured with an embryo

**Fig 7:**
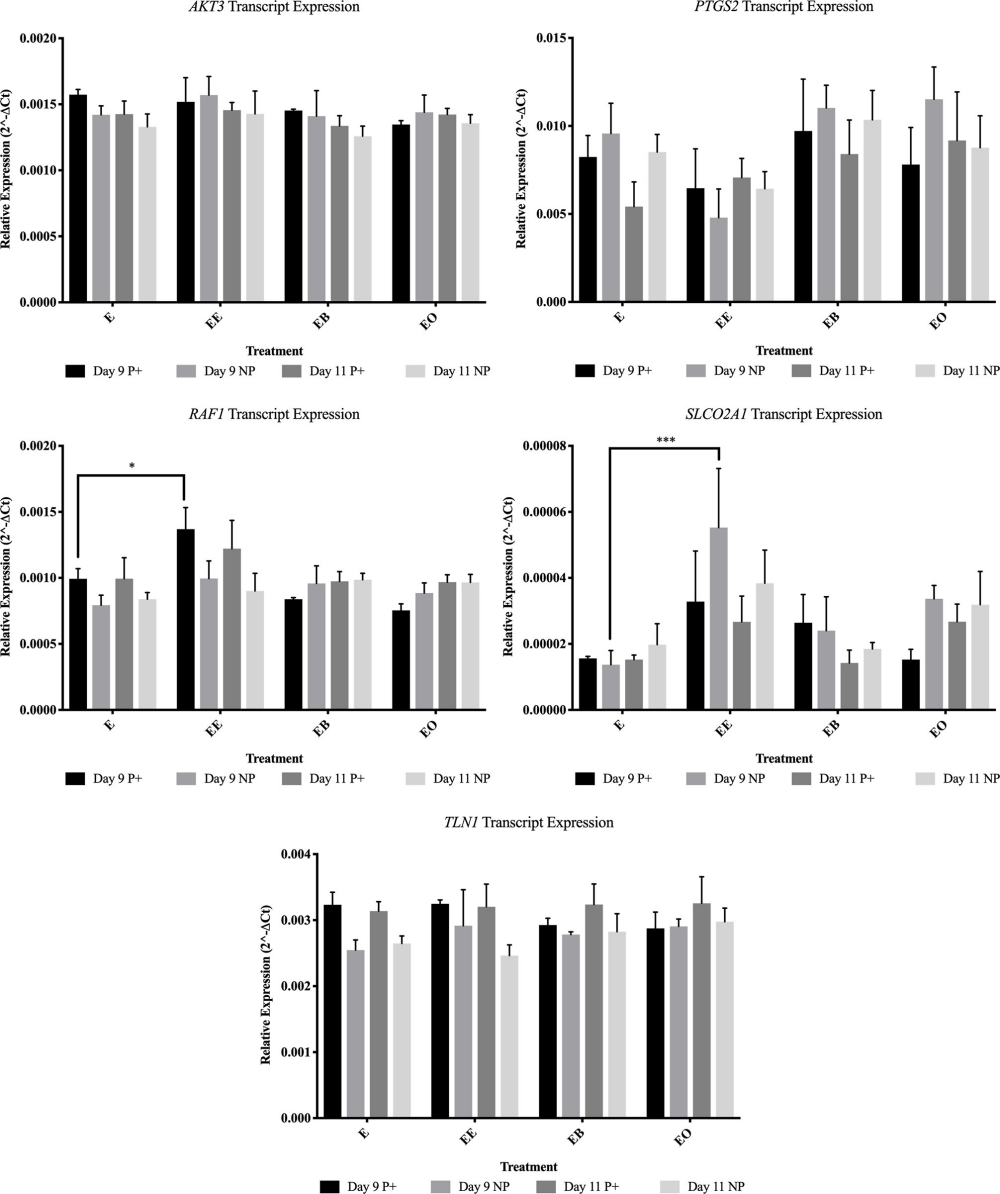
Transcript abundance levels for AKT3, PTGS2, RAF1, SLCO2A1 and TLN1 for all treatments in endometrial samples from pregnant (P+) and non-pregnant (NP) mares. This figure contains the gene expression data for AKT3, PTGS2, RAF1, SLCO2A1 and TLN1. E = no treatment (control); EE = endometrium with an embryo in contact with the luminal side of the biopsy sample; EB = endometrium with beads in contact with the luminal side of the biopsy sample; EO = endometrium with beads in contact with the luminal side of the biopsy sample. * = *P* ≤ 0.05, ** = *P* ≤ 0.01 and *** = *P* ≤ 0.001. Graphs are mean ± SEM.

**Fig 8:**
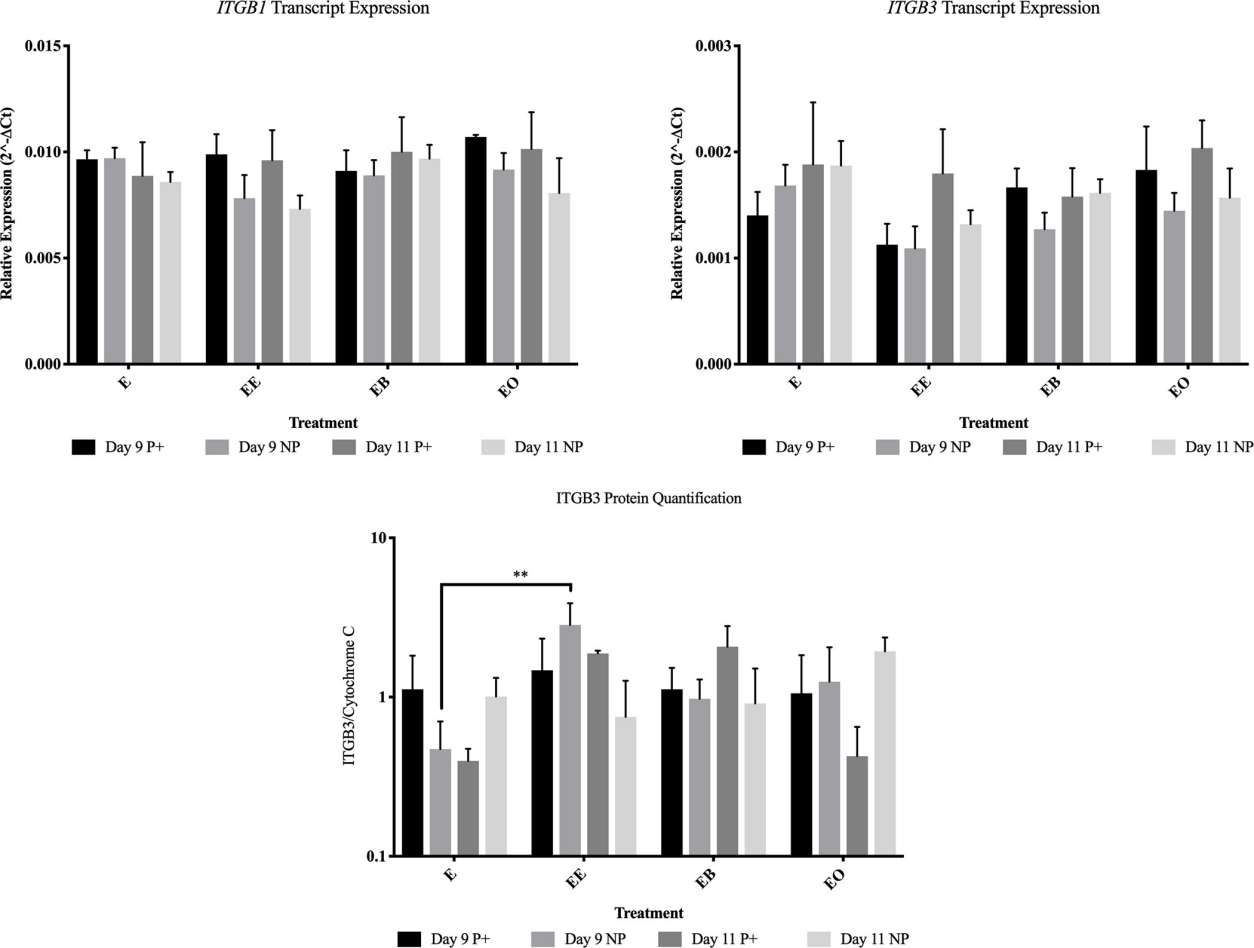
Gene and protein abundance for β-integrins for all treatments in endometrial samples from pregnant (P+) and non-pregnant (NP) mares. This figure contains all of the protein and gene expression data that was analyzed in this project. E = no treatment (control); EE = endometrium with an embryo in contact with the luminal side of the biopsy sample; EB = endometrium with beads in contact with the luminal side of the biopsy sample; EO = endometrium with beads in contact with the luminal side of the biopsy sample. * = *P* ≤ 0.05, ** = *P* ≤ 0.01 and *** = *P* ≤ 0.001. Graphs are mean ± SEM.

On day 9 both transcript and protein changes were identified based upon the presence of an embryo. Only one gene, *RAF1*, was more abundant (*P* = 0.047) in endometrial samples co-cultured with an embryo from samples from pregnant mares (Fig 7). *BCL-2* (*P* = 0.004), *ITGA4* (*P* = 0.028) and *SLCO2A1* (*P* = 0.001) were all higher in abundance levels in samples from non-pregnant mares after culture with an embryo for 24 hours (Figs 3, 4 and 7). In samples from pregnant mares, α-ACTININ (*P* = 0.002), CAV1 (*P* = 0.006) and CCND1 (*P* = 0.002) were more abundant in control samples (Figs 3 and 5). ITGB3 was more abundant (*P* = 0.004) in the presence of an embryo in samples from non-pregnant mares (Fig 8).

On day 11, no transcripts were changed due to the presence of an embryo in samples from pregnant mares. In samples from non-pregnant mares, *ACTN2* (*P* = 0.050) was the only gene that was higher in abundance in the presence of an embryo (Fig 5). *ACTN1* (*P* = 0.007), *ACTN4* (*P* = 0.005), *CAV1* (*P* < 0.001) and *ITGA6* (*P* = 0.035) were all more abundant in control samples from non-pregnant mares (Figs 3–5). Only one protein, BCL-2, was more abundant (*P* = 0.009) in samples from pregnant mares in the presence of an embryo (Fig 3). PAK6 was the only protein more abundant (*P* = 0.047) in samples from non-pregnant mares in the presence of an embryo (Fig 3).

### PGF secretion after co-culture

**Fig 9:**
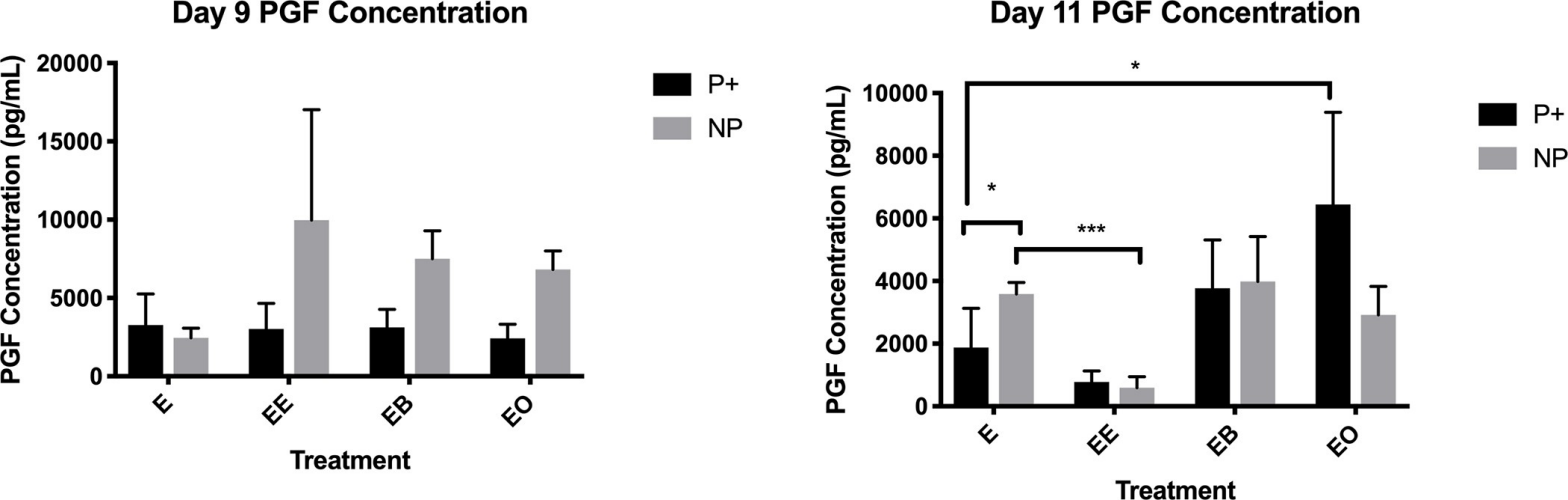
PGF concentration in the medium after 24 hours of co-culture for all treatments for endometrial samples from pregnant (P+) and non-pregnant (NP) mares. This is a graphical representation of the concentration of PGF in the medium after 24 hours of culture with the specific treatment. E- = no treatment (control); EE = endometrium with an embryo in contact with the luminal side of the biopsy sample; EB = endometrium with beads in contact with the luminal side of the biopsy sample; EO = endometrium with beads in contact with the luminal side of the biopsy sample. * = *P* ≤ 0.05, ** = *P* ≤ 0.01 and *** = *P* ≤ 0.001. PGF concentration is in pg/mL, mean ± SEM.

PGF concentration in the medium was evaluated to determine if the presence of beads, oil or an embryo would alter PGF secretion. On day 9 there was no change in PGF secretion due to any of the treatments. On day 11, the presence of oil increased PGF secretion in samples from pregnant mares compared to control samples (*P* = 0.043). Most importantly, in samples from non-pregnant mares, the presence of an embryo decreased PGF secretion (*P* = 0.003) compared to the sample that was not in the presence of an embryo (591.73 pg/mL versus 3282.96 pg/mL respectively). Interestingly, when comparing samples from pregnant and non-pregnant mares after 24 hours of culture without the presence of an embryo, PGF secretion was lower in samples obtained from pregnant mares (*P* = 0.036). Fig 9 shows the concentration of PGF in each treatment group.

## Discussion

Previous studies reported that focal adhesions play an important role at the maternal-fetal interface in many species, but none have identified their potential role in equine endometrium [21]. While it is known that focal adhesions are present in equine endometrium, their impact on maternal recognition of pregnancy and PGF secretion have not been studied to date [20]. In the present study, we evaluated the presence of specific focal adhesion molecules in equine endometrium that were immediately snap frozen after collection, but also after the sample had been challenged for 24 hours with one of four treatments (an embryo, beads/mechanical force, peanut oil, or with no contact). The genes tested in this study were derived from previous studies in our laboratory and studies on focal adhesions in other species [20,21]. Others have hypothesized that placing a glass bead in the uterus of a mare will extend luteal function [25]. This suggested that MRP is triggered based upon the presence of a round object moving throughout the uterine body. Even though this finding is highly disputed, the beads used in the present study mimicked the contact of a glass marble on endometrium [16]. The peanut oil was used because previous studies have indicated that infusing a mare’s uterus with oil will prolong diestrous [15].

Mechanical force on the endometrium (beads) induced changes in focal adhesion molecule abundance. This could be due to the fact that focal adhesion molecules are mechanical sensors, so just the contact of something on these molecules will induce a change but may not illicit a response [21]. Studies have shown that focal adhesions and their downstream effects are dependent upon the composition and rigidity of the ECM [26,27]. Therefore, while the beads are making contact and causing a change in the focal adhesions, they may not be eliciting a true response. This lack of response was further validated when the PGF concentration was measured in the medium. On both days 9 and 11, the PGF concentration in the medium was similar between samples with and without bead co-culture. The beads actually resulted in a larger amount of PGF (*P* = 0.004) being secreted into the medium than the embryo (3987.4 pg/mL and 591.7 pg/mL respectively) on day 11 in samples from non-pregnant and pregnant mares (*P* = 0.019; 3771.9 pg./mL and 775.8 pg/mL respectively). This data shows *in vitro* that the presence of beads making physical contact with the endometrium is not enough to signal maternal recognition of pregnancy, ultimately leading to a decrease in PGF secretion.

Contrary to previous reports, this study showed that the application of peanut oil on endometrial samples does not cause a decrease in PGF secretion [15]. This was true for both days 9 and 11. Interestingly, on day 9, no FAMs were changed based upon the presence of peanut oil on endometrial samples from non-pregnant mares and *PAK6* was the only transcript more abundant (*P* = 0.012) in samples from pregnant mares. α-ACTININ (*P* = 0.026), CAV1 (*P* = 0.024) and CCND1 (*P* = 0.005) were all decreased due to the presence of peanut oil on day 9 in samples from pregnant mares. On day 11, only *ACTN4* (*P* = 0.011) and *CAV1* (*P* = 0.001) were decreased due to the presence of peanut oil and BCL-2 was more abundant in samples from pregnant mares. Peanut oil actually caused the endometrium from pregnant mares on day 11 to secrete more PGF compared to the control (6444.4 pg/mL and 3586.0 pg/mL respectively).

The presence of an embryo altered many FAMs and PGF secretion. It was expected that samples from non-pregnant mares would be altered in the presence of an embryo, but samples from pregnant mares would not. This is because once focal adhesion molecules are activated, they can stay active due to internal contractility in cells [28]. It was hypothesized that once the endometrial samples from pregnant mares were exposed to an embryo in utero, even once the embryo was removed for culture, the focal adhesions would remain active for the 24 hour culture. On day 9, samples from both pregnant and non-pregnant mares were altered by the presence of an embryo. Only *RAF1* was more abundant (*P* = 0.047) in samples from pregnant mares due to the presence of an embryo. α-ACTININ (*P* = 0.002), CAV1 (*P* = 0.006) and CCND1 (*P* = 0.002) were less abundant in samples from pregnant mares. In samples from non-pregnant mares on day 9, *BCL-2* (*P* = 0.004), *ITGA4* (*P* = 0.028), *SLCO2A1* (*P* = 0.001) and ITGB3 (*P* = 0.004) were more abundant due to the presence of an embryo. Even with these alterations, on day 9 there was no change in PGF secretion in the presence of an embryo. It is thought that although the embryo is mobile, it does not reach maximum mobility until days 11–14, therefore the signaling has not occurred for maternal recognition of pregnancy and there is no change in PGF secretion [4].

On day 11, only BCL-2 was more abundant (*P* = 0.009) due to the presence of an embryo in samples from pregnant mares. All other alterations occurred in samples from non-pregnant mares. *ACTN1* (*P* = 0.007), *ACTN4* (*P* = 0.005), *CAV1* (*P* < 0.001) and *ITGA6* (*P* = 0.035) were less abundant in the presence of an embryo and *ACTN2* (*P* = 0.050) and PAK6 (*P* = 0.047) were more abundant in the presence of an embryo in samples from non-pregnant mares. PGF secretion was dramatically decreased (*P* = 0.003) in samples from non-pregnant mares in the presence of an embryo (591.7 pg/mL versus 3586.0 pg/mL) on day 11. Another interesting observation was when comparing the PGF secretion in control samples between pregnant and non-pregnant mares, although the sample from a pregnant mare was not in the presence an embryo for 24 hours, the amount of PGF secreted remained lower than the samples from non-pregnant mares (1877.8 pg/mL and 3586.0 pg/mL respectively). These data further validate the idea that once the machinery is activated, it remains active for a period of time without external pressure [28].

An interesting observation from this dataset was that α-ACTININ, CAV1, and CCND1 were altered due to all treatments. These three proteins may play a crucial role in sensing force in the extracellular matrix and relaying that to the cell. Caveolin-1 (CAV1) is a mechano-mediator that has been identified in rat endometrium and increases during pregnancy [29]. It is located apically in uterine epithelial cells and human Ishikawa cells [30]. α-ACTININ is a Ca^+2^ sensitive actin filament cross-linking protein [31]. It is a cytoskeletal protein, and in contrast to actin, is located specifically on the apical plasma membrane of rat uterine epithelial cells [32]. It was hypothesized to be involved in actin filament reorganization during early pregnancy, especially the period of receptivity [32]. Cyclin D1 (CCND1) plays an important role in the progression of the cell cycle [33]. CCND1 acts as an oncogene in many different human neoplasia’s when overexpressed [34]. More specifically, CCND1 overexpression has been reported in many endometrial carcinomas [35]. Overall, these three proteins can be playing a distinct role in sensing and reacting to external stimuli from the extracellular matrix but are not transducing the signal into the cell to signal maternal recognition of pregnancy leading to a decrease in PGF.

**Fig 10:**
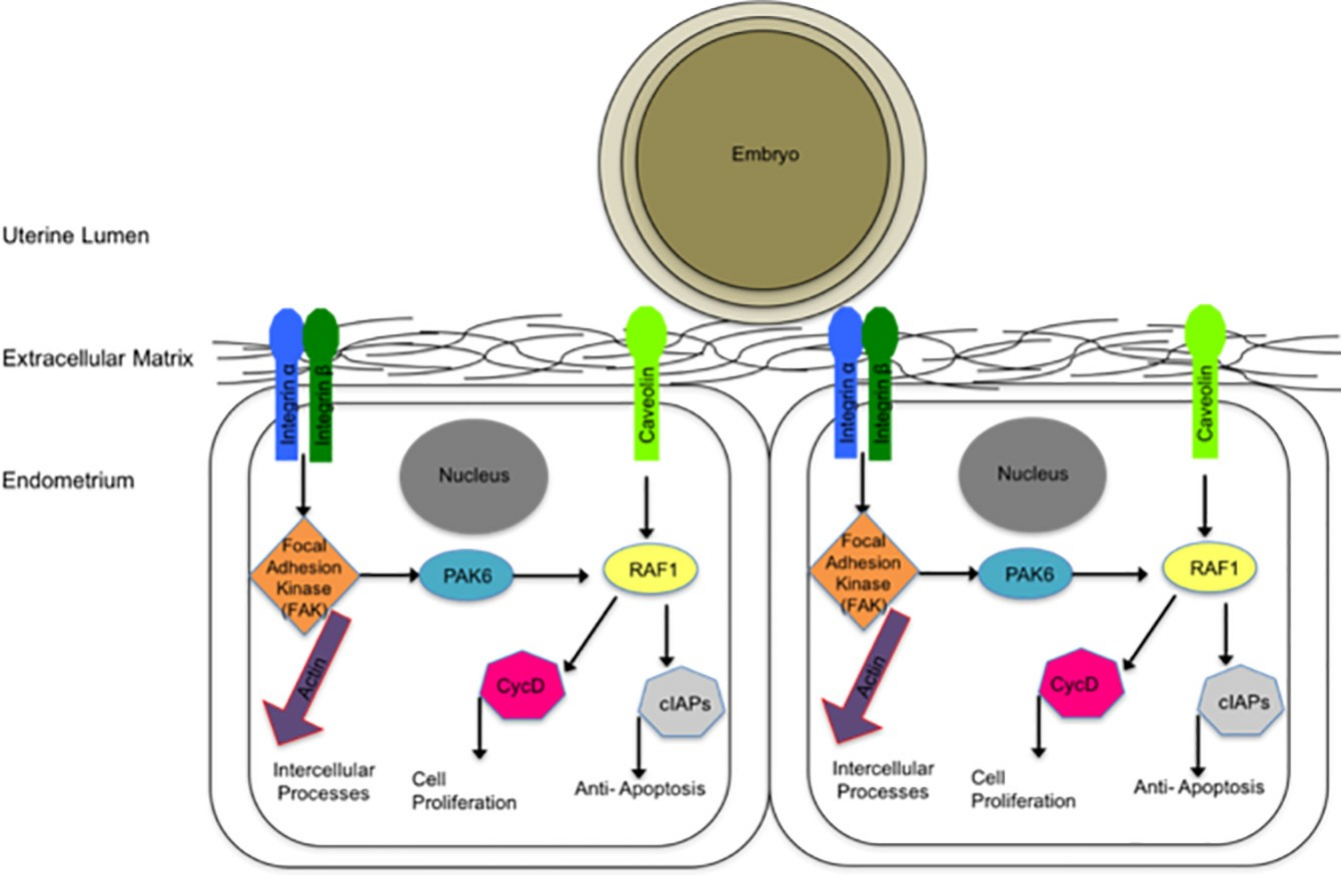
A schematic of a mobile embryo and focal adhesions within the endometrium. As the embryo is being bounced around the uterine lumen from days 9–16 it is causing the assembly and activation of focal adhesions. Once activated, these focal adhesions intracellularly can be impacting multiple processes, including the prevention of PGF release.

In conclusion, we determined that the contact of beads, peanut oil, and an embryo cause changes in focal adhesion molecules in endometrium from pregnant and non-pregnant mares. In contrast, the contact with an embryo on day 11 on endometrial samples from non-pregnant mares was the only treatment capable of decreasing PGF secretion. These results suggest the contact of an embryo alone, and only for 24 hours, is enough to alter focal adhesions and decrease PGF secretion. Based upon this data we hypothesize that a mobile embryo in the uterus activates focal adhesions, which lead to a decrease in PGF secretion (Fig 10). Future studies will need to evaluate what portion of the embryo is responsible for this change in PGF secretion and the mechanism by which that message is being relayed to the endometrial cell.

## Supporting information

S1 Table Designed primer sequences and amplicon length for each focal adhesion gene. (DOCX)
