# Supplementary material for: The role of embryo contact and focal adhesions during maternal recognition of pregnancy
Source: PLoS One. 2019 Mar 5;14(3):e0213322. doi: 10.1371/journal.pone.0213322 (PMC6400379; doi:10.1371/journal.pone.0213322)
Supplement: S1 Table — (DOCX) [file pone.0213322.s001.docx]

**S1 Table. Designed PCR Primers for Focal Adhesions**

| Gene | Forward Primer (5'-3') | Reverse Primer (5'-3') |
| --- | --- | --- |
| *ACTN1* | TGACCCAGAAGCGGAGGGAA | CCCTCCATCCAGTTGTTGAAGGG |
| *ACTN2* | CTGACCGAGGGAAAATGCGA | CCAGCGTCATTTTCACATTGCCA |
| *ACTN3* | GGAGGAGTACGAGAAGCTTGCCA | AGTCCCGAAAGTCCTCCAGCT |
| *ACTN4* | GAGAACATCGACGAGGACTTCC | CAGCGCCTTGTTCACATTGTTGA |
| *AKT3* | TGGACAGAAGCTATCCAGGCTGT | TGGAAGCATCCATCTCTTCCTC |
| *BCL2* | GACGCTTTGCCACGGTAGTG | ATCTCCCGGTTGACGCTCTC |
| *CAV1* | GGCAACATCTACAAGCCCAACA | TTGAGATGCTTGGGGTCGCG |
| *CCND1* | CGTGAAGTTCATTTCCAACCCGC | TGTGAGGCGGTGGTAGGACA |
| *ITGA4* | TCAACATCACAGTGCATGCCG | CCATGAACAGCTAGCATGACTTCA |
| *ITGA5* | AGGGAGAGGAGCCTGTGGAA | AGCGGCTCCTTTTCTGTGCG |
| *ITGA6* | TCCCGGCCTGTGATTAACATT | CAGGCCTTAACCTTGAGGCATATC |
| *ITGA10* | ACCCACGGCTGAATTTGGAT | CCCCTACAGGGCAGCGATAA |
| *ITGAV* | GGTCGCTGCCACTGACATTA | CGAAGCCCTTTGCAGAGACA |
| *ITGAX* | CCAGCCAACAACCTTCCGCA | TCACACTGGTAAAGGCCGCC |
| *ITGB1* | AGTCTTGGAACCGATCTGATGAATGA | TGCAAGGGTTTCTGAGCTTGGC |
| *ITGB3* | AGGAGGTGAAGAAGCAGAGCGT | GGATGCATCATTCCTCCAGCCA |
| *PAK6* | GGCTCCTGAAGTGATCTCCAGGT | TGGCTTGCACTGGTGAGTCG |
| *PTGS2* | CCTTTTCCAACCTCTCGTATTACACCA | TCCACAATCTCTTTTGAATCAGGAAGC |
| *FAK* | AGATCCTCTCCCCAGTGTACAGA | GTTGCAGCCCTTGTCCGTGA |
| *RAF1* | CTAAAACCCCTGTGCCGGCA | TGGAGAGCATCACTTCACTCGCT |
| *SLCO2A1* | TGAACGGGGCAAGGAAGATGGA | TGGGCCAGGACTGTCAGCAT |
| *TLN1* | TGAAGACGATGCAGTTTGAGCCG | TGACAGAAACAGCCCAAAGTCGC |
| *ACTB* | CGACATCCGTAAGGACCTGT | CAGGGCTGTGATCTCCTTCT |
| *B2M* | GTGTTCCGAAGGTTCAGGTT | ATTTCAATCTCAGGCGGATG |
| *GAPDH* | AGAAGGAGAAAGGCCCTCAG | GGAAACTGTGGAGGTCAGGA |
| *RN18S* | AACGACACTCTGGCATGCTAACTA | CGCCACTTGTCCCTCTAAGAA |
